# *Panax notoginseng* Attenuates Bleomycin-Induced Pulmonary Fibrosis in Mice

**DOI:** 10.1155/2011/404761

**Published:** 2011-03-06

**Authors:** Kuen-Daw Tsai, Shu-Mei Yang, Jen-Chih Lee, Ho-Yiu Wong, Chuen-Ming Shih, Ting-Hui Lin, Min-Jen Tseng, Wei Chen

**Affiliations:** ^1^Department of Internal Medicine, China Medical University Beigang Hospital, Yunlin 651, Taiwan; ^2^Institute of Molecular Biology, National Chung Cheng University, Chiayi 62102, Taiwan; ^3^Department of Chinese Medicine, China Medical University Beigang Hospital, Yunlin 651, Taiwan; ^4^Department of Respiratory Therapy, China Medical University, Taichung 40402, Taiwan; ^5^College of Medical Science and Technology, Chung Shan Medical University, Taichung 40204, Taiwan; ^6^Division of Pulmonary and Critical Care Medicine, Chia-Yi Christian Hospital, Chiayi 600, Taiwan; ^7^Department of Life Sciences, National, Chung Hsing University, Taichung 402, Taiwan

## Abstract

*Panax notoginseng* (PN) is a traditional Chinese herb experimentally proven to have anti-inflammatory effects, and it is used clinically for the treatment of atherosclerosis, cerebral infarction, and cerebral ischemia. This study aimed to determine the anti-inflammatory effects of PN against bleomycin-induced pulmonary fibrosis in mice. First, in an in vitro study, culture media containing lipopolysaccharide (LPS) was used to stimulate macrophage cells (RAW 264.7 cell line). TNF-*α* and IL-6 levels were then determined before and after treatment with PN extract. In an animal model (C57BL/6 mice), a single dose of PN (0.5 mg/kg) was administered orally on Day 2 or Day 7 postbleomycin treatment. The results showed that TNF-*α* and IL-6 levels increased in the culture media of LPS-stimulated macrophage cells, and this effect was significantly inhibited in a concentration-dependent manner by PN extract. Histopathologic examination revealed that PN administered on Day 7 postbleomycin treatment significantly decreased inflammatory cell infiltrates, fibrosis scores, and TNF-*α*, TGF-*β*, IL-1*β*, and IL-6 levels in bronchoalveolar lavage fluid when compared with PN given on Day 2 postbleomycin treatment. These results suggest that PN administered in the early fibrotic stage can attenuate pulmonary fibrosis in an animal model of idiopathic pulmonary fibrosis.

## 1. Introduction

Idiopathic pulmonary fibrosis (IPF) is a fatal disease characterized by progressive and irreversible lung fibrosis, without any known effective therapy [1]. Although associated molecular mechanisms remain poorly understood, hallmark pathological features include fibroblastic foci representing focal areas of active fibrogenesis, fibroblast replication, and extracellular matrix (ECM) deposition. Myofibroblasts, by secreting ECM molecules, which include fibrillar collagens, fibronectin, elastin, and proteoglycans, play an important role in the pathogenesis of pulmonary fibrosis [2, 3]. Various theories are posited to explain this process, such as the production of fibroblast chemotactic factors by activated alveolar epithelial cells and the release of growth factors involved in reepithelization, such as transforming growth factor beta (TGF-*β*), platelet-derived growth factor (PDGF), insulin-like growth factor-1 (ILGF-1), and endothelin-1 (ET-1) [4–7]. Inflammatory cytokines, including tumor necrosis factor (TNF) and interleukin (IL), are also important in the development of pulmonary fibrosis [8]. 

*Panax notoginseng* (PN) is a highly valued traditional Chinese medicine that reportedly has a variety of pharmacologic activities. Clinically, PN is used in the treatment of acute ischemic stroke [9] and cardiovascular disease [10]. In experimental studies, its documented pharmacological effects include anti-inflammatory, haemostatic, antioxidant, hypolipidemic, hepatoprotective, renoprotective, and estrogen-like activities [11]. A variety of studies have also shown PN to have anti-inflammatory activity, probably via COX-2, toll-like receptor, or nuclear factor kappa B (NF-*κ*B) signaling pathways [12–17]. Moreover, PN may inhibit hepatic or renal fibrosis when these organs are damaged [18–21]. Studies addressing the anti-inflammatory effect of PN on lung disease are quite limited, and only one report shows that PN attenuates acute lung injury induced by intestinal ischemia/reperfusion in rats [22].

Previous studies have shown that blocking TGF-*β* and TNF-*α* may attenuate the development of pulmonary fibrosis [23, 24]. Accordingly, this study hypothesized that PN inhibits pulmonary fibrosis by suppressing TNF-*α* and TGF-*β* [19, 25] with the aim of evaluating in a murine model its protective effects on bleomycin-induced pulmonary fibrosis in terms of survival rate, lung tissue histology, fibrosis score, and inflammatory cytokines. 

## 2. Materials and Methods

### 2.1. Preparation of *Panax notoginseng* (PN) Extract

PN was purchased from Fu Tan Pharmaceutical Co., Ltd, Taiwan in 2006. Air-dried flowers (50 g) of PN were cut into small pieces and extracted with 80% methanol in a reflux condenser for 5 h. The methanol extract of PN was filtered through Whatman No. 1 paper and concentrated in a vacuum evaporator (18.74 g; 37.48% yield). The lyophilized powder was dissolved in culture-grade dimethyl sulfoxide (DMSO; Sigma-Aldrich, St. Louis, MO, USA) prior to use and then sterile-filtered through a 0.45 *μ*M Millipore filter. 

### 2.2. Cell Culture

Macrophages (RAW 264.7 cell line) were kept in an atmosphere of 95% air and 5% CO_2_ and incubated in Dulbecco's modified Eagle's medium (DMEM; GibcoBRL, Grand Island, NY, USA) supplemented with 5% heat-inactivated fetal bovine serum (FBS; Hyclone, Logan, UT, USA), 100 g/mL streptomycin, and 100 U/mL penicillin (GibcoBRL). Cells were maintained via weekly passage, and cell viability was determined by a 3-(4,5-dimethyl-thiazol-2-yl)-2,5-diphenyl tetrazolium bromide (MTT; Sigma) reduction assay. The cells used for experimentation were at 60%–80% confluence.

Briefly, cells were preincubated overnight in 24-well plates at a density of 2 × 10^5^ cells per well and then washed with phosphate-buffered saline (PBS). Cells with various concentrations of PN extract were treated with lipopolysaccharide (LPS) (*Escherichia coli *055:B5, Sigma-Aldrich) for 24 h and grown in media containing MTT (0.5 mg/mL) at 37°C for 4 h. After removing the culture supernatants, the resulting dark blue crystals were dissolved with DMSO. Absorbance values were read at 550 nm on an automated SpectraMAX 340 (Molecular Devices, Sunnyvale, CA, USA). All determinations were confirmed by at least three independent experiments.

The culture supernatants collected from treated cells were used to determine the inhibitory effects of PN extract on the production of TNF-*α* and IL-6 as measured by enzyme-linked immunosorbent assay (ELISA). The samples were analyzed according to the manufacturer's recommendations with mouse cytokine-specific DuoSet ELISA Development System (R&D Systems, Minneapolis, MN, USA). Concentrations of TNF-*α* and IL-6 were calculated according to a standard curve generated using each of the recombinant cytokines in the ELISA kits. 

### 2.3. Animals and Experimental Protocols

Healthy male C57BL/6 mice weighing 200 to 250 g from the experimental research center of China Medical University (Taichung, Taiwan) were housed in a specific cage with a 12-h light/dark cycle and with free access to water and food ad libitum. The institutional committee for animal care approved all procedures involving animals. To examine the safety dosage, 0.5 mg/kg, 1 mg/kg, and 2 mg/kg of PN were administered orally to the mice. No mice died during the 14-day administration of 0.5 mg/kg PN, while the 14-day mortality rate was 67% for mice administered 2 mg/kg PN. Thus, 0.5 mg/kg PN was used as the experimental dose administered via oral gavage.

**Figure 1:**
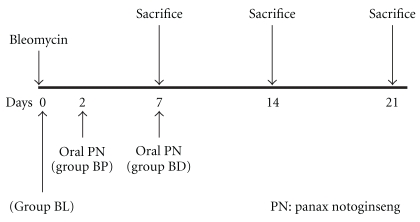
Experimental protocol for bleomycin-induced pulmonary fibrosis and administration of *Panax notoginseng* (PN) at different time points.

For instillation, animals were first anesthetized with ether, and bleomycin (Aventis, Brussels, Belgium) suspended in sterile NaCl 0.9% at 1 U/kg was then instilled transorally into the animals' lungs via the trachea. Control mice (*n* = 6) were instilled with a corresponding volume of NaCl 0.9% (NS group). The remaining animals were divided randomly into the following three groups: (1) the BL group included mice (*n* = 6) instilled with bleomycin; (2) the BP group included mice (*n* = 6) instilled with bleomycin and PN was administered orally on Day 2 post-bleomycin instillation; and (3) the BD group included mice (*n* = 6) instilled with bleomycin and PN was administered orally on Day 7 post-bleomycin instillation (Figure 1). The mice in the BL and BP groups were sacrificed on Days 7, 14, or 21 after treatment, whereas the mice in the BD group were sacrificed only on Day 14 or 21 because PN was administered on Day 7 post-bleomycin instillation. 

### 2.4. Histopathologic Analysis

After sacrifice, the right lower lobe of the lung from each animal was harvested and fixed in 10% formalin and embedded in paraffin after 48 h. Ten consecutive longitudinal sections (4 *μ*m thick) of the lungs were stained with hematoxylin and eosin (H&E stain). The resulting slides were examined under light microscope at a photodocumentation facility. 

To grade the extent of lung fibrosis, each successive field was individually assessed for the severity of interstitial fibrosis using the semiquantitative grading system as previously described [26]. The entire lung section was reviewed under ×100 magnification. For each of the 30 to 35 microscopic fields that were reviewed, a score ranging from 0 (normal lung) to 8 (total fibrosis) was assigned. Criteria for grading pulmonary fibrosis were as follows: Grade 0, normal lung; Grade 1, minimal fibrous thickening of alveolar or bronchial walls; Grades 2-3, moderate thickening of walls without obvious damage to lung architecture; Grades 4-5, increased fibrosis with definite damage to lung architecture and formation of fibrous bands or small fibrous mass; Grades 6-7, severe distortion of structure and large fibrous areas; “honeycomb lung” was also placed in this category; and Grade 8, total fibrotic obliteration of the field. The mean score of all fields was taken as the fibrosis score of that lung section.

To assess the bronchoalveolar lavage fluid (BALF), the right main bronchus was clamped and bronchoalveolar lavage of the left lung was performed three times with 2 mL normal saline solution. BALF was pooled and centrifuged at 1200 g for 10 min, and the supernatant was harvested for cytokine analysis. BALF levels of TGF-*β*, TNF-*α*, IL-1*β* and IL-6 were quantified using ELISA specific for the previously mentioned mouse cytokines (Biosource International, Nivelles, Belgium). 

### 2.5. Statistics

All statistical analyses were performed using a commercial statistical software (SPSS for Windows, version 15.0, Chicago, IL, USA). Data are presented as the mean plus or minus the standard error of the mean of three samples and are representative of three independent experiments. Differences between two means were analyzed by Student's *t*-test. Data sets with multiple comparisons were evaluated by one-way analysis of variance (ANOVA) with Dunnett's post test. Values of *P* < .05 were deemed to be statistically significant. 

## 3. Results

### 3.1. In Vitro Study

**Figure 2:**
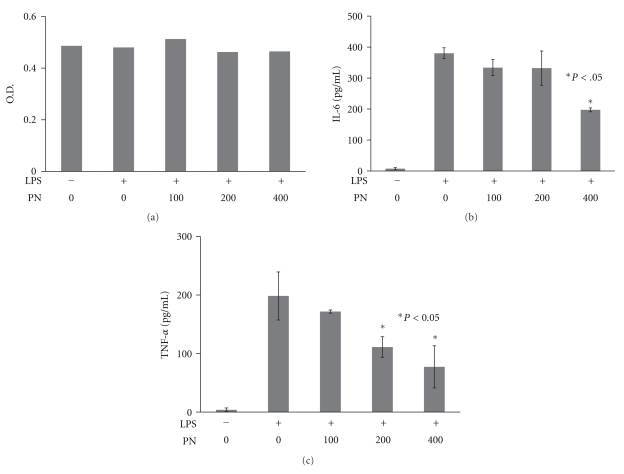
PN extract inhibited LPS-induced inflammatory cytokines in macrophage cells. (a) MTT assay to evaluate cell viability at various concentrations of PN. (b) IL-6 levels increased in the culture media of LPS-stimulated macrophage cells and significantly decreased with 400 *μ*g/mL of PN. (c) The levels of TNF-*α* were significantly inhibited in a concentration-dependent manner by treatment with PN extract. Data are presented as means ± SEM. (PN unit: *μ*g/mL).

The MTT assay was used to evaluate cell viability at various concentrations of PN (100, 200, and 400 *μ*g/mL). The OD of the various PN concentrations showed no significant difference (0.51, 0.46, and 0.47, resp.) (Figure 2(a)). Further investigation of the anti-inflammatory effects of PN on LPS-stimulated macrophages using ELISA showed that TNF-*α* and IL-6 levels were increased. At different concentrations of PN (100, 200, and 400 *μ*g/mL), the corresponding levels of IL-6 were 333.68, 331.88, and 197.40 pg/mL, respectively. At PN 400 *μ*g/mL, the levels of IL-6 were significantly reduced (*P* < .05) (Figure 2(b)). At increasing concentrations of PN (100, 200, and 400 *μ*g/mL), the levels of TNF-*α* were 171.73, 111.14, and 77.22 pg/mL, respectively. Thus, treatment with PN extract significantly reduced TNF-*α* levels in a concentration-dependent manner (Figure 2(c)). 

### 3.2. Lung Histology and Pulmonary Fibrosis Score

**Figure 3:**
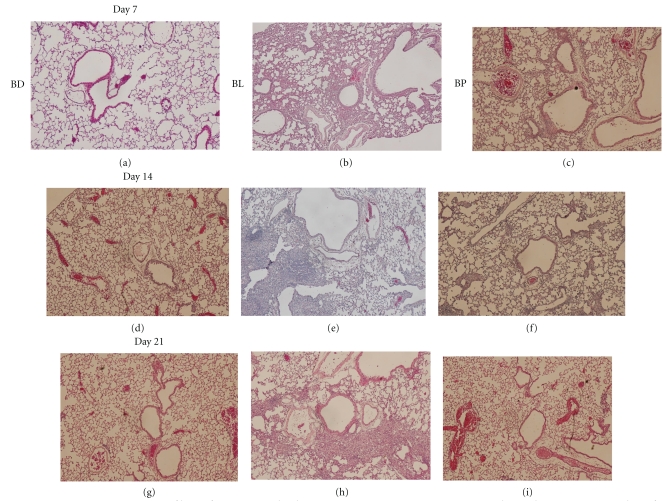
Representative sections of lungs from mice in the three treatment groups. Lung tissues were obtained on Days 7, 14, and 21 after instillation of bleomycin and physiologic saline (H&E stain, ×100 magnification).

Histopathologic examination of H&E-stained lung tissue sections showed an obvious inflammatory response on Day 7 post-bleomycin instillation, and the lungs progressed to fibrosis on Day 14, which became even more severe on Day 21. The lung parenchyma of the bleomycin-treated group (BL group) showed severe epithelial degeneration, inflammatory cell infiltration, fibrosis, vascular congestion, and disturbance of alveolar structure (Figures 3(b), 3(e), and 3(h)), while the PN-treated bleomycin group (BP group) showed moderate inflammatory cell infiltration, and mild epithelial degeneration, fibrosis, vascular congestion, and disturbance of alveolar structure (Figures 3(c), 3(f), and 3(i)).

**Figure 4:**
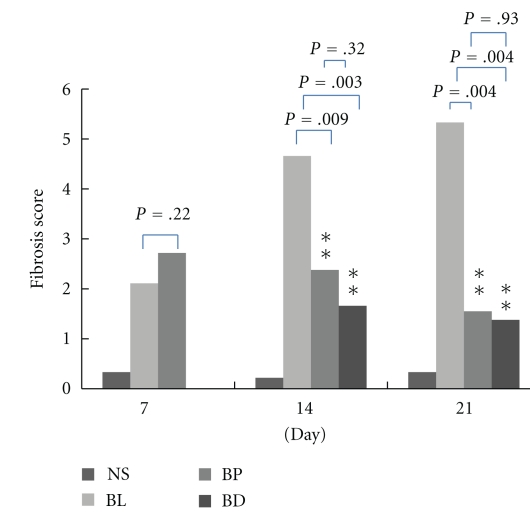
Pulmonary fibrosis scores of the different groups on Days 7, 14, and 21 post-bleomycin treatment. Data are presented as means ± SEM.

Using Fast Green stain, the severity of interstitial fibrosis was assessed using a semiquantitative grading system [26]. Lung tissues from the bleomycin-treated group (BL group) exhibited significant bleomycin-induced lung injury. As shown in Figure 4, pulmonary fibrosis scores were higher in the BP group than in the BL group, although not statistically significant (*P* = .22). In contrast to Day 7, the pulmonary fibrosis scores of the BP group were significantly lower than those of the BL group on Days 14 and 21 (*P* = .009 and  .004, resp.). In the BD group, the pulmonary fibrosis scores were significantly reduced on Days 14 and 21. 

### 3.3. Proinflammatory Cytokines in BALF

**Figure 5:**
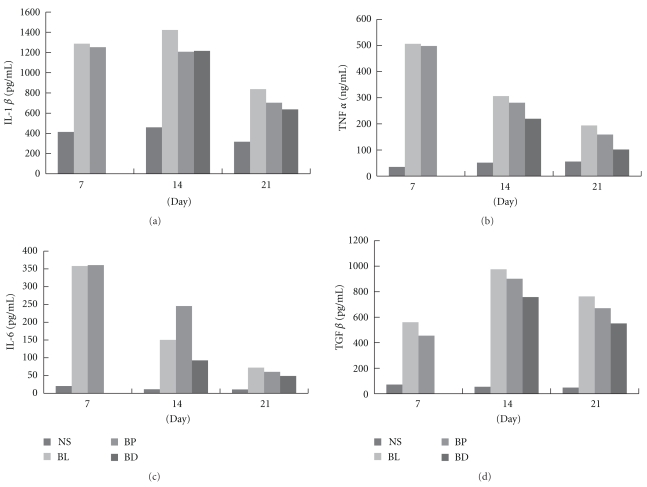
(a) IL-1*β*, (b) TNF-*α*, (c) IL-6, and (d) TGF-*β* levels in bronchoalveolar lavage fluid (BALF) of mice treated with saline (NS), bleomycin (BL), PN since Day 2 post-bleomycin treatment (BP), or PN since Day 7 post-bleomycin treatment (BD). Data are presented as means ± SEM (*n* = 6 for each group).

In the BL group, levels of TGF-*β*, TNF-*α*, IL-1*β*, and IL-6 in BALF were significantly increased on Days 7, 14, and 21 after bleomycin injection, but the levels of these cytokines were significantly reduced in the BD group (bleomycin with PN-Day 7) compared with the BL group on Days 14 and 21 (Figure 5). In contrast to the BP group (bleomycin with PN-Day 2), TGF-*β* levels were significantly increased on Day 7, similar to levels of the BL group but higher on Day 14. 

## 4. Discussion

Idiopathic pulmonary fibrosis (IPF) is a progressive and fatal pulmonary disease without proven drug therapies [4]. Its clinical features vary, with differences in age at onset, presentation, and clinical course [27]. This study shows that PN administered on Day 7 post-bleomycin treatment results in significant reductions in pulmonary fibrosis scores and cytokines in mice, indicating that PN can be an effective treatment in the early stages of this disease.

Although the pathogenesis of IPF is not well clarified, recent studies have focused on dysregulated epithelial-mesenchymal interactions, an imbalance in T(H)1/T(H)2 cytokine profile, and a potential role for aberrant angiogenesis [4]. Proinflammatory cytokines like TNF-*α*, IL-1*β*, IL-6, and TGF-*β* might play important roles [28, 29]. In the BALF of bleomycin-treated mice, TNF-*α*, IL-1*β*, IL-6, and TGF-*β* levels of BALF were significantly increased. Although the mechanisms by which PN attenuates lung fibrosis are unclear, this study shows that TNF-*α* and IL-6 cytokine levels are significantly reduced by PN in alveolar macrophage culture. In the BALF of mice, TNF-*α*, IL-1*β*, IL-6, and TGF-*β* levels were significantly reduced by PN. It is possible that PN inhibits early phase TNF-*α* production by enhancing NO production, to cause cellular damage via anti-inflammatory effects [19, 30]. In a similar manner, PN can ameliorate the effects of the fibrotic process in the renal interstitium and block the tubular epithelial-myofibroblast transdifferentiation [31, 32]. 

In addition, soluble IL-1*β* bioactivity and autocrine IL-1*β*-dependent IL-6 up-regulation are critical initiators of fibroblast activation and proliferation that likely play a role in the fibroproliferative response in human acute lung injury [33]. In contrast to the BP group (PN started on Day 2), IL-6 levels and pulmonary fibrosis scores were lower in the BD group. It is likely that early administration of PN may stimulate the activation of fibro-proliferation by activating IL-6. 

In the dosage of PN administration, three concentration levels were administered to animals initially (0.5, 1.0, and 2.0 mg/kg/day). The mortality rate of C57BL/6 mice was 57% for the 7-day course with 2 mg/kg/day of PN. In contrast, no mice died with a dose of 0.5 mg/kg/day. To further evaluate the pathologic changes in dead mice with 2 mg/kg/day of PN, there is a severe inflammatory response in the alveolus and lung parenchyma, with hyaline membrane change. Features of histopathology are quite similar to those in acute respiratory distress syndrome (ARDS). As a result, the dose of 0.5 mg/kg/day of PN was used as the experimental dosage in the animal model. 

## 5. Conclusions

In summary, PN administered in the early fibrotic stage, not the inflammatory stage, can treat pulmonary fibrosis induced by bleomycin. 

## References

[1] Gross TJ, Hunninghake GW (2001). Idiopathic pulmonary fibrosis. *New England Journal of Medicine*.

[2] Zhang K, Rekhter MD, Gordon D, Phan SH (1994). Myofibroblasts and their role in lung collagen gene expression during pulmonary fibrosis: a combined immunohistochemical and in situ hybridization study. *American Journal of Pathology*.

[3] Kuhn C, McDonald JA (1991). The roles of the myofibroblast in idiopathic pulmonary fibrosis: ultrastructural and immunohistochemical features of sites of active extracellular matrix synthesis. *American Journal of Pathology*.

[4] Horowitz JC, Thannickal VJ (2006). Idiopathic pulmonary fibrosis: new concepts in pathogenesis and implications for drug therapy. *Treatments in Respiratory Medicine*.

[5] Selman M, King TE, Pardo A (2001). Idiopathic pulmonary fibrosis: prevailing and evolving hypotheses about its pathogenesis and implications for therapy. *Annals of Internal Medicine*.

[6] Horowitz JC, Thannickal VJ (2006). Epithelial-mesenchymal interactions in pulmonary fibrosis. *Seminars in Respiratory and Critical Care Medicine*.

[7] Selman M, Pardo A (2003). The epithelial/fibroblastic pathway in the pathogenesis of idiopathic pulmonary fibrosis: tying loose ends. *American Journal of Respiratory Cell and Molecular Biology*.

[8] Phan SH, Kunkel SL (1992). Lung cytokine production in bleomycin-induced pulmonary fibrosis. *Experimental Lung Research*.

[9] Chen X, Zhou M, Li Q (2008). Sanchi for acute ischaemic stroke. *Cochrane Database of Systematic Reviews*.

[10] Chan P, Thomas GN, Tomlinson B (2002). Protective effects of trilinolein extracted from Panax notoginseng against cardiovascular disease. *Acta Pharmacologica Sinica*.

[11] Ng TB (2006). Pharmacological activity of sanchi ginseng (Panax notoginseng). *Journal of Pharmacy and Pharmacology*.

[12] Rhule A, Navarro S, Smith JR, Shepherd DM (2006). Panax notoginseng attenuates LPS-induced pro-inflammatory mediators in RAW264.7 cells. *Journal of Ethnopharmacology*.

[13] Chang SH, Choi Y, Park JA (2007). Anti-inflammatory effects of BT-201, an n-butanol extract of Panax notoginseng, observed in vitro and in a collagen-induced arthritis model. *Clinical Nutrition*.

[14] Rhule A, Rase B, Smith JR, Shepherd DM (2008). Toll-like receptor ligand-induced activation of murine DC2.4 cells is attenuated by Panax notoginseng. *Journal of Ethnopharmacology*.

[15] Zhang YG, Zhang HG, Zhang GY (2008). Panax notoginseng saponins attenuate atherosclerosis in rats by regulating the blood lipid profile and an anti-inflammatory action. *Clinical and Experimental Pharmacology and Physiology*.

[16] Jung HW, Seo UK, Kim JH, Leem KH, Park YK (2009). Flower extract of Panax notoginseng attenuates lipopolysaccharide-induced inflammatory response via blocking of NF-*κ*B signaling pathway in murine macrophages. *Journal of Ethnopharmacology*.

[17] Wan JB, Lee SMY, Wang JD (2009). Panax notoginseng reduces atherosclerotic lesions in ApoE-deficient mice and inhibits TNF-*α*-induced endothelial adhesion molecule expression and monocyte adhesion. *Journal of Agricultural and Food Chemistry*.

[18] Xie XS, Yang M, Liu HC (2008). Influence of ginsenoside Rg1, a panaxatriol saponin from Panax notoginseng, on renal fibrosis in rats with unilateral ureteral obstruction. *Journal of Zhejiang University B*.

[19] Park WH, Lee SK, Kim CH (2005). A Korean herbal medicine, Panax notoginseng, prevents liver fibrosis and hepatic microvascular dysfunction in rats. *Life Sciences*.

[20] Peng XD, Dai LL, Huang CQ, He CM, Yang B, Chen LJ (2009). Relationship between anti-fibrotic effect of Panax notoginseng saponins and serum cytokines in rat hepatic fibrosis. *Biochemical and Biophysical Research Communications*.

[21] Geng J, Peng W, Huang Y, Fan H, Li S (2010). Ginsenoside-Rg1 from Panax notoginseng prevents hepatic fibrosis induced by thioacetamide in rats. *European Journal of Pharmacology*.

[22] Rong L, Chen Y, He M, Zhou X (2009). Panax notoginseng saponins attenuate acute lung injury induced by intestinal ischaemia/reperfusion in rats. *Respirology*.

[23] de Rochemonteix-Galve B, Dayer JM, Junod AF (1990). Fibroblast-alveolar cell interactions in sarcoidosis and idiopathic pulmonary fibrosis: evidence for stimulatory and inhibitory cytokine production by alveolar cells. *European Respiratory Journal*.

[24] Kollias G, Douni E, Kassiotis G, Kontoyiannis D (1999). The function of tumour necrosis factor and receptors in models of multi-organ inflammation, rheumatoid arthritis, multiple sclerosis and inflammatory bowel disease. *Annals of the Rheumatic Diseases*.

[25] Zhang HS, Wang SQ (2006). Notoginsenoside R1 from Panax notoginseng inhibits TNF-*α*-induced PAI-1 production in human aortic smooth muscle cells. *Vascular Pharmacology*.

[26] Ashcroft T, Simpson JM, Timbrell V (1988). Simple method of estimating severity of pulmonary fibrosis on a numerical scale. *Journal of Clinical Pathology*.

[27] Katzenstein ALA, Myers JL (1998). Idiopathic pulmonary fibrosis: clinical relevance of pathologic classification. *American Journal of Respiratory and Critical Care Medicine*.

[28] Tsoutsou PG, Gourgoulianis KI, Petinaki E (2006). Cytokine levels in the sera of patients with idiopathic pulmonary fibrosis. *Respiratory Medicine*.

[29] Agostini C, Gurrieri C (2006). Chemokine/cytokine cocktail in idiopathic pulmonary fibrosis. *Proceedings of the American Thoracic Society*.

[30] Wu F, Zhang SS, Kang GF (2003). Effects of panax notoginseng saponins on the expression of tumor necrosis factor alpha and secretion phospholipase A2 in rats with liver fibrosis. *Zhonghua Gan Zang Bing Za Zhi*.

[31] Su BH, Li Z, Fan JM, Wang M, Tang R (2005). Effects of panax notoginseng saponins on the process of renal interstitial fibrosis after unilateral ureteral obstruction in rats. *Journal of Sichuan University*.

[32] Wei Y, Fan JM, Pan LP (2002). Effect of Panax notoginseng saponins on human kidney fibroblast. *Zhongguo Zhong Xi Yi Jie He Za Zhi*.

[33] Olman MA, White KE, Ware LB (2004). Pulmonary edema fluid from patients with early lung injury stimulates fibroblast proliferation through IL-1*β*-induced IL-6 expression. *Journal of Immunology*.

